# Efficacy and Safety of the iStent infinite Trabecular Micro-Bypass Device for Glaucoma: A Systematic Review and Meta-Analysis

**DOI:** 10.31486/toj.26.0015

**Published:** 2026

**Authors:** Ryan Fischer, Christopher Duncombe, Chinwendu Okolie, Lily Caglianone

**Affiliations:** ^1^ St. John Fisher University, Rochester, NY; ^2^ International Association of Providers of AIDS Care, Washington, DC; ^3^ Independent Data Scientist, Enugu, Nigeria; ^4^ Rush University, Chicago, IL

**Keywords:** *Glaucoma*, *glaucoma drainage implants*, *glaucoma–open-angle*, *intraocular pressure*, *microsurgery*, *minimally invasive surgical procedures*, *ophthalmologic surgical procedures*, *prosthesis implantation*

## Abstract

**Background:**

The iStent infinite (Glaukos Corporation) is a third-generation trabecular micro-bypass device approved for stand-alone use in patients with inadequately controlled open-angle glaucoma, but overall efficacy and safety estimates have not been quantitatively synthesized via meta-analysis. Our objective was to evaluate the 12-month efficacy and safety of iStent infinite device implantation by examining intraocular pressure reduction and glaucoma medication reduction.

**Methods:**

We conducted a systematic review and meta-analysis per the Preferred Reporting Items for Systematic reviews and Meta-Analyses (PRISMA) 2020 guidelines after registering the review protocol with the International Prospective Register of Systematic Reviews (PROSPERO). We searched PubMed/MEDLINE, Cochrane Central Register of Controlled Trials (CENTRAL), and ClinicalTrials.gov from inception through November 2025 for studies reporting 12-month outcomes after iStent infinite device implantation (stand-alone or combined with phacoemulsification). Pooled mean changes in intraocular pressure and medications were calculated, heterogeneity was assessed with I^2^, and surgical approach (stand-alone vs combined with phacoemulsification) was evaluated via meta-regression.

**Results:**

Three nonrandomized studies comprising 199 eyes with 12-month follow-up were included. The pooled mean intraocular pressure reduction at 12 months was 4.48 mm Hg (95% CI 3.04 to 5.92; *P*<0.0001) with high heterogeneity (I^2^=83.4%). Meta-regression showed greater intraocular pressure reduction with stand-alone implantation vs phacoemulsification-combined procedures (an additional 2.16 mm Hg; 95% CI 0.52 to 3.81; *P*=0.010), explaining 88.4% of heterogeneity. The pooled mean reduction in glaucoma medications was 0.48 (95% CI 0.29 to 0.67; *P*<0.0001) with moderate heterogeneity (I^2^=45.0%) and no significant difference by surgical approach (*P*=0.65).

**Conclusion:**

iStent infinite device implantation was associated with clinically meaningful 12-month reductions in intraocular pressure, a modest reduction in medication burden, and a favorable short-term safety profile.

## INTRODUCTION

Glaucoma is a leading cause of irreversible blindness, globally affecting an estimated 76 million individuals aged 40 to 80 years, with projections approaching 111.8 million by 2040.^1,2^ Primary open-angle glaucoma accounts for the majority of cases, with an estimated global prevalence of approximately 3.54%.^1^ Disease progression results in optic nerve damage and visual field loss, ultimately leading to irreversible blindness if left untreated.^3^

Elevated intraocular pressure is the primary modifiable risk factor for glaucoma progression, and lowering intraocular pressure has been consistently shown to reduce the risk of optic nerve damage and visual field loss.^4-6^ The Early Manifest Glaucoma Trial found that each 1 mm Hg reduction in intraocular pressure was associated with an approximate 10% decreased risk of glaucoma progression.^4^ The Advanced Glaucoma Intervention Study found that eyes with intraocular pressure consistently <18 mm Hg experienced minimal visual field deterioration.^7^

The traditional therapeutic approach to glaucoma management is via intraocular pressure reduction and follows a stepwise approach, beginning with topical medications, advancing to laser trabeculoplasty, and ultimately requiring incisional surgery when conservative measures fail.^8-10^ However, each treatment modality has limitations. Initial management with topical intraocular pressure–lowering agents is frequently limited by poor adherence. Studies have shown nonadherence rates ranging from 5% to 80%, with substantial discontinuation over time, including nearly half of patients stopping therapy within 6 months.^11,12^ Common barriers to medication adherence include forgetfulness, ocular and systemic side effects, financial burden, and difficulties with drop administration.^13^

Traditional glaucoma surgeries such as trabeculectomy and tube shunt procedures carry the risk of vision-threatening complications. The Tube Versus Trabeculectomy study reported early complication rates of 37% for trabeculectomy and 21% for tube shunt procedures.^14^ Early postoperative complications include choroidal effusion and wound leak; a less frequent later complication is endophthalmitis.^14^

Micro-invasive glaucoma surgery has emerged as an alternative treatment option.^15-17^ According to the American Glaucoma Society, micro-invasive glaucoma surgery is a group of surgical procedures that improve aqueous humor outflow through ab interno approaches with minimal disruption to the sclera and conjunctiva, thereby preserving tissue for future surgical interventions.^18^ Micro-invasive glaucoma surgery procedures have 5 key characteristics: an ab interno approach, minimal trauma to target tissue, high safety profile, modest intraocular pressure–lowering efficacy, and rapid recovery.^15^

**Figure 1.**
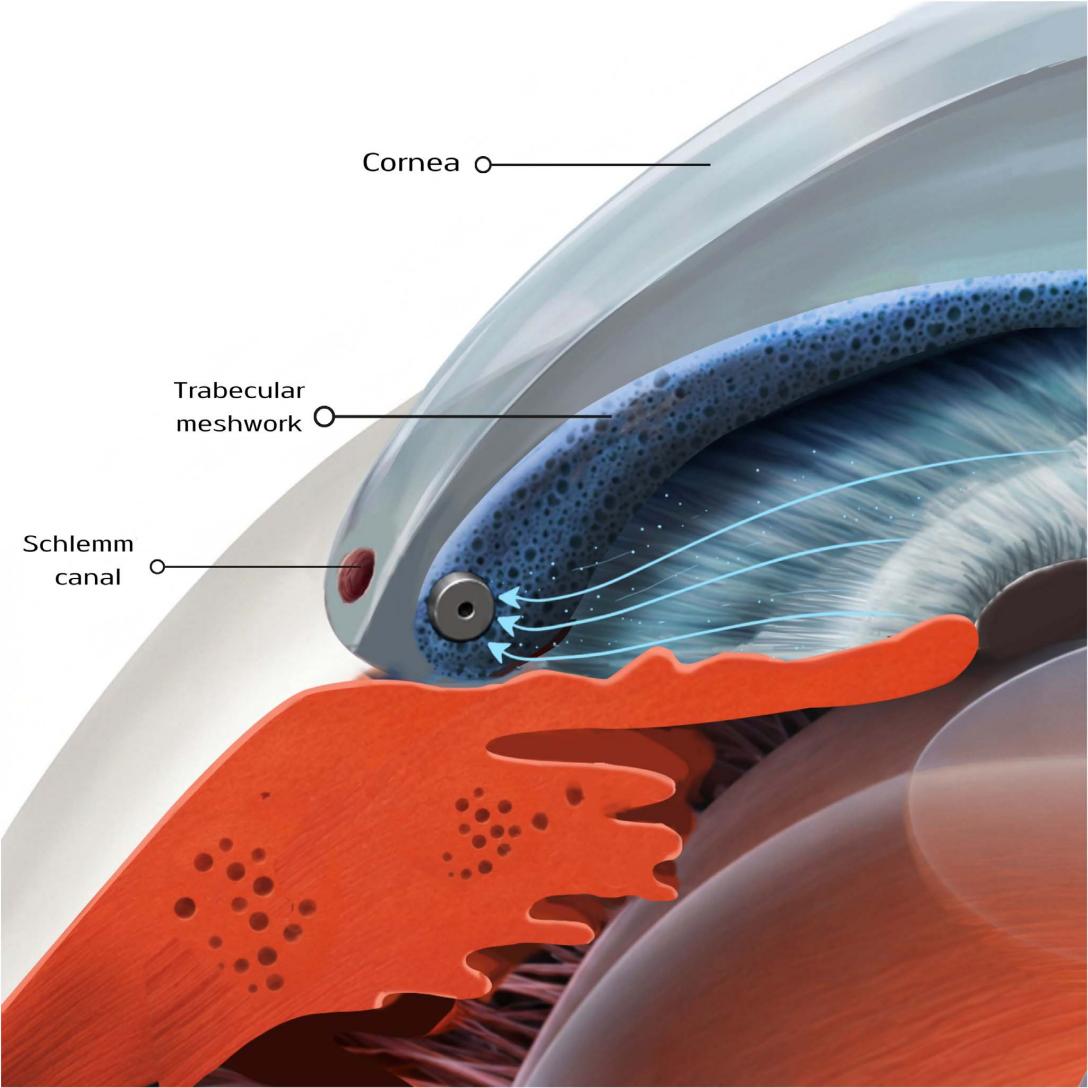
The iStent infinite device bypasses the trabecular meshwork and directs aqueous flow through the stent and into the Schlemm canal. In this custom illustration, the iStent infinite device is enlarged for clarity.

A trabecular micro-bypass stent is a micro-invasive glaucoma surgery device that reduces intraocular pressure by creating direct communication between the anterior chamber of the eye and the Schlemm canal (Figure 1),^19^ targeting the conventional aqueous outflow pathway responsible for the majority of aqueous humor drainage in the healthy eye.^20,21^ The juxtacanalicular tissue of the trabecular meshwork at the inner wall of the Schlemm canal is the primary site of aqueous outflow resistance.^20^ Trabecular micro-bypass stents bypass this resistance point by creating a direct channel that allows flow from the anterior chamber into the collector channel system.

The iStent family of trabecular micro-bypass devices (Glaukos Corporation) first received US Food and Drug Administration (FDA) approval in 2012 based on trial data showing superior intraocular pressure reduction when stent implantation was combined with cataract surgery vs cataract surgery alone.^22^ The second-generation iStent featured 2 preloaded stents and was also used in conjunction with cataract surgery.^23^

The iStent infinite, a third-generation device that received FDA 510(k) clearance in August 2022, is the first trabecular micro-bypass stent cleared for stand-alone use without concurrent cataract surgery in patients with primary open-angle glaucoma that is inadequately controlled with medical or surgical therapy.^24,25^ The device consists of 3 heparin-coated titanium stents preloaded into a single-use autoinjector system. Each stent measures 360 μm in length with a 360-μm flange diameter and contains multiple lateral outlets of lumens of varying diameters (four 50-μm and two 80-μm lumens).^26^ The 3-stent configuration covers up to 240 degrees of the trabecular meshwork, providing access to multiple collector channel systems while occupying only approximately 3% of the trabecular meshwork circumference.^25,26^

Since the iStent infinite device received FDA clearance, clinical evidence has been provided for both stand-alone and phacoemulsification-combined use. Despite emerging data, no systematic review with meta-analysis has synthesized the available evidence to provide pooled estimates of efficacy and safety outcomes. Such an analysis is needed to inform clinical decision-making, establish realistic expectations for patients and surgeons, identify knowledge gaps, and guide future research directions. The objective of this systematic review and meta-analysis was to evaluate the efficacy and safety of the iStent infinite trabecular micro-bypass device for the treatment of glaucoma. Our primary outcomes were intraocular pressure reduction and glaucoma medication burden reduction at 12 months following iStent infinite device implantation. Our secondary outcome was adverse events associated with the procedure.

## METHODS

We conducted this systematic review and meta-analysis in accordance with the Preferred Reporting Items for Systematic reviews and Meta-Analyses (PRISMA) 2020 guidelines^27^ after registering the review protocol with the International Prospective Register of Systematic Reviews (PROSPERO; registration number CRD420251253395).

### Search Strategy

We searched the following electronic databases from inception through November 2025 to find all relevant studies evaluating the iStent infinite trabecular micro-bypass device for glaucoma treatment: PubMed/MEDLINE, Cochrane Central Register of Controlled Trials (CENTRAL), and ClinicalTrials.gov. Keywords used in the search strategy included “iStent infinite” OR “iStent third generation” or “iStent” or “third-generation trabecular micro-bypass” OR “three-stent trabecular micro-bypass” OR “iStent iS3” AND “glaucoma.” We did not apply any language restrictions, but only English language studies were found.

### Eligibility Criteria and Study Selection

Studies were included if they met the following criteria: (1) evaluated iStent infinite trabecular micro-bypass device implantation either as a stand-alone procedure or combined with phacoemulsification (micro-invasive cataract surgery); (2) included patients with any form of glaucoma or ocular hypertension; (3) reported quantitative outcomes for intraocular pressure and/or glaucoma medication use; (4) provided follow-up data for 12 months; and (5) were published as full-text articles or were available as complete clinical trial reports.

Studies were excluded if they (1) were case reports or case series with <10 eyes; (2) lacked primary outcome data on intraocular pressure or medication reduction; (3) were conference abstracts without full-text publications; or (4) included mixed micro-invasive glaucoma surgery devices without separate outcome reporting for the iStent infinite device.

Two reviewers screened all retrieved records to identify potentially eligible studies. Full-text articles were obtained for all studies that met the initial screening criteria or when eligibility could not be determined from the abstract alone. One reviewer independently assessed full-text articles against the eligibility criteria, with a second reviewer verifying all selections. Disagreements were resolved through discussion. The study selection process was documented using a PRISMA flow diagram.

### Data Extraction

The following information was extracted: (1) study characteristics (first author, year of publication, journal, study design, study length, total eyes enrolled, eyes remaining at 12-month follow-up); (2) patient demographics and baseline characteristics (mean age with standard deviation and glaucoma severity when reported); (3) baseline and 12-month intraocular pressure data (mean intraocular pressure for the full cohort and the 12-month cohort); (4) medication burden (baseline and 12-month mean number of glaucoma medications for the full cohort and the 12-month cohort); and (5) safety outcomes (device removal, endophthalmitis/infection, serious device-related adverse events, and secondary glaucoma interventions following iStent infinite device implantation).

The proportions of eyes achieving intraocular pressure thresholds of ≤12 mm Hg, ≤15 mm Hg, and ≤18 mm Hg were extracted when reported but were not consistently available across studies and therefore are not included in the pooled analysis.

Medication burden was defined as the mean number of glaucoma medications used per patient. Medication change was calculated as the difference between the mean number of medications at baseline and at 12 months, expressed as the mean number of medications per patient. The proportions of medicated eyes at baseline and at 12 months were extracted when reported; however, these data were not consistently available across studies and therefore are not included in the pooled analysis. When data were not clearly stated in the manuscript, attempts were made to derive values from the available information (eg, simple subtractions).

### Statistical Analysis and Risk of Bias

Meta-analyses were performed using random-effects models^28^ implemented in the metafor package in the R statistical software (The R Foundation). Random-effects models were selected a priori given the anticipated clinical and methodological heterogeneity across nonrandomized studies differing in design, population, and procedure type. For continuous outcomes (mean intraocular pressure change, mean medication change), mean differences with 95% confidence intervals (CIs) were calculated. For studies not reporting standard deviation of change from baseline, the standard deviation was derived using correlation-based methods assuming a correlation coefficient of 0.5.

Statistical heterogeneity was assessed using the I^2^ statistic, which represents the percentage of variability in effect estimates attributable to between-study heterogeneity rather than sampling error.^29^ I^2^ values of 25%, 50%, and 75% were interpreted as low, moderate, and high heterogeneity, respectively. The Cochran Q statistic (Q) was also calculated to test for statistical heterogeneity. When substantial heterogeneity was detected, potential sources were explored through subgroup (stand-alone vs combined with phacoemulsification) meta-regression analyses.

Subgroup analyses were planned a priori to examine the impact of surgical approach (stand-alone iStent infinite device implantation vs combined with phacoemulsification) using meta-regression. Statistical significance of subgroup differences was assessed using the test for moderators in the meta-regression framework.

Publication bias assessment using funnel plots and the Egger regression test was not performed because of the small number of included studies (n=3), as these methods require a minimum of 10 studies for meaningful interpretation.^30^

All statistical analyses were performed using R with the metafor package, with 2-sided *P* values <0.05 considered statistically significant. Forest plots were generated to display individual study results and pooled estimates with 95% CIs.

Risk of bias for each study was assessed using the Risk of Bias In Non-randomized Studies–of Interventions (ROBINS-I) tool. The ROBINS-I tool evaluates bias across 7 domains: confounding, selection of participants, classification of interventions, deviations from intended interventions, missing data, measurement of outcomes, and selection of the reported results. Each domain is rated as having a low, moderate, serious, or critical risk of bias.^31^

For this meta-analysis, we followed the guidance provided in the *Cochrane Handbook for Systematic Reviews of Interventions*.^32^

## RESULTS

**Figure 2.**
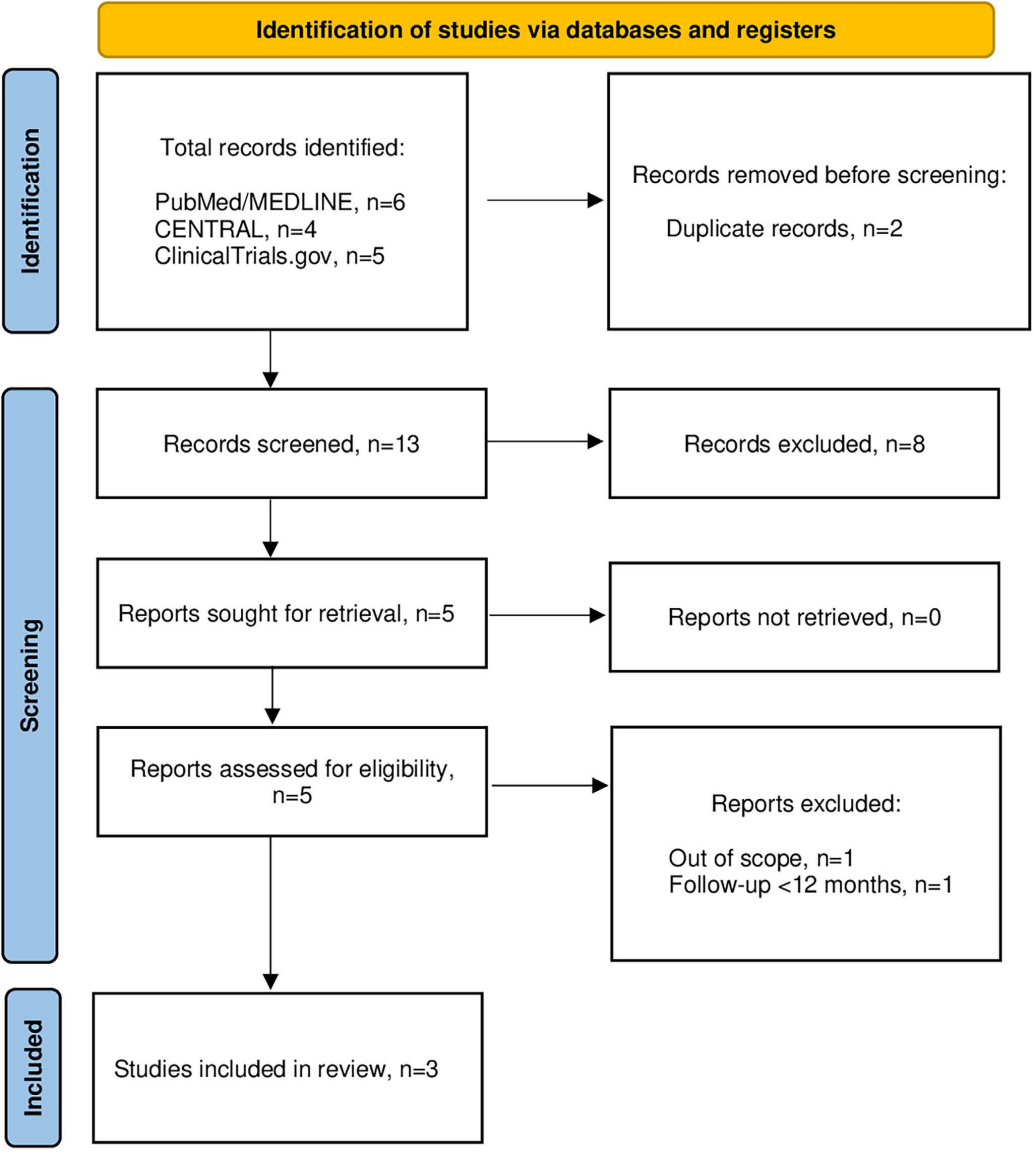
**Preferred Reporting Items for Systematic reviews and Meta-Analyses (PRISMA) flow diagram of study selection.** CENTRAL, Cochrane Central Register of Controlled Trials.

Our systematic literature search identified 13 records. Following title and abstract screening, 5 full-text articles were assessed for eligibility. Three studies met all inclusion criteria and were included in the quantitative analysis. The PRISMA flow diagram is presented in Figure 2.

### Study Designs and Baseline Characteristics

The 3 included studies examined a combined 426 eyes at baseline and 199 eyes at 12-month follow-up: Sarkisian et al (2023; n=71),^25^ Vest et al (2025; n=32),^33^ and Shultz et al (2025; n=96).^24^ Study characteristics are summarized in the Table. All 3 studies were nonrandomized: 1 prospective multicenter single-arm FDA pivotal trial^25^ and 2 retrospective real-world case series.^24,33^ Sarkisian et al^25^ evaluated stand-alone iStent infinite device implantation in patients with open-angle glaucoma uncontrolled by surgical or medical therapy, while Vest et al^33^ and Shultz et al^24^ examined the iStent infinite device combined with phacoemulsification in patients with mild to moderate primary open-angle glaucoma and cataract.

**Table.** Designs, Characteristics, and Primary Outcomes of Studies Evaluating the iStent infinite Trabecular Micro-Bypass Device (Glaukos Corporation)

	Study		
Variable	Sarkisian et al, 2023^25^	Vest et al, 2025^33^	Shultz et al, 2025^24^
Study design	Prospective, multicenter, single-arm, open-label FDA pivotal trial	Retrospective, consecutive, real-world case series (single surgeon)	Retrospective, multicenter, consecutive case series
Procedure type	Stand-alone	Phacoemulsification-combined	Phacoemulsification-combined
Patient age, years, mean ± SD	71.9 ± 8.4	73.8 ± 6.7	73.3 ± 7.4
Number of eyes at 12 months	71	32	96
Glaucoma severity, %			
Mild	NR	66.1	62.2
Moderate	NR	33.9	34.4
Severe	NR	0	2.8
Intraocular pressure, mm Hg, mean ± SD			
At baseline	23.4 ± 2.8	18.1 ± 3.3	17.2 ± 4.2
At 12 months	17.5 ± NR	13.8 ± 3.4	13.8 ± 3.0
Number of medications, mean ± SD			
At baseline	3.1 ± 0.9	1.38 ± 0.91	1.24 ± 0.91
At 12 months	2.7 ± 1.0	1.06 ± 1.13	0.61 ± 0.96

Notes: Sarkisian et al^25^ enrolled 72 eyes total; 71 eyes had 12-month follow-up data. Baseline intraocular pressure and medication values for Vest et al^33^ and Shultz et al^24^ reflect the consistent cohort (eyes with 12-month follow-up data) rather than the full enrolled cohort, consistent with the approach used in each study.

FDA, US Food and Drug Administration; NR, not reported.

Mean baseline intraocular pressure ranged from 17.2 to 23.4 mm Hg, with Sarkisian et al^25^ enrolling a more refractory population (mean 23.4 ± 2.8 mm Hg) compared to the phacoemulsification-combined studies (Vest et al,^33^ 18.1 ± 3.3 mm Hg; Shultz et al,^24^ 17.2 ± 4.2 mm Hg). Mean baseline medication burden ranged from 1.24 to 3.1 medications, with the stand-alone cohort again demonstrating higher baseline values. Disease severity also differed. Vest et al^33^ reported that 66.1% of patients had mild glaucoma and 33.9% had moderate glaucoma. Similarly, almost all patients had mild to moderate glaucoma in the Shultz et al^24^ study. While severity distribution was not explicitly reported in Sarkisian et al,^25^ the refractory nature of the population suggests advanced disease.

### Risk of Bias Assessment

The risk of bias assessment with the ROBINS-I tool^31^ revealed that 1 study had a moderate overall risk of bias and 2 studies had a serious overall risk of bias. All 3 studies demonstrated a low risk of bias in measurement of outcomes. Sarkisian et al^25^ demonstrated a moderate risk of bias across the domains of confounding, selection of participants, missing data, and selection of the reported results, with a low risk of bias for classification of interventions, deviations from intended interventions, and measurement of outcomes. The 2 retrospective studies (Vest et al^33^ and Shultz et al^24^) showed a serious risk of bias in the domains of confounding, selection of participants, and missing data, primarily attributable to the studies’ retrospective designs and potential for unmeasured confounding.

### Primary Outcome: Intraocular Pressure Reduction

**Figure 3.**
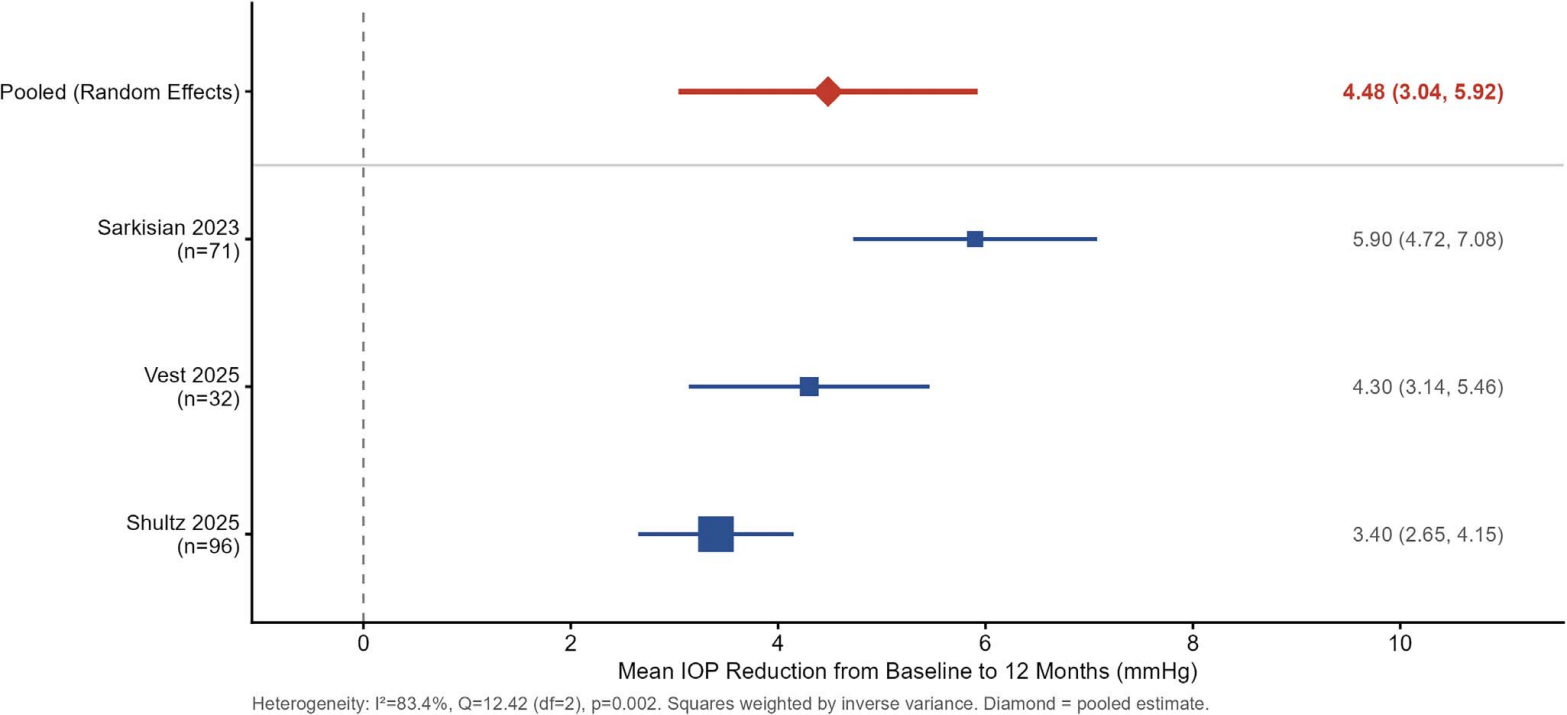
Forest plot of mean intraocular pressure (IOP) reduction from baseline to 12 months following iStent infinite device implantation in 3 studies.^24,25,33^

Meta-analysis of the 3 studies (n=199 eyes) demonstrated a pooled mean intraocular pressure reduction of 4.48 mm Hg (95% CI 3.04 to 5.92; *P*<0.0001) at 12 months postimplantation (Figure 3). Individual study estimates ranged from 3.40 mm Hg in Shultz et al^24^ to 5.90 mm Hg in Sarkisian et al.^25^ Statistical heterogeneity was high (I^2^=83.4%; Q=12.42; *P*=0.002), with substantial variability in treatment effects across studies.

**Figure 4.**
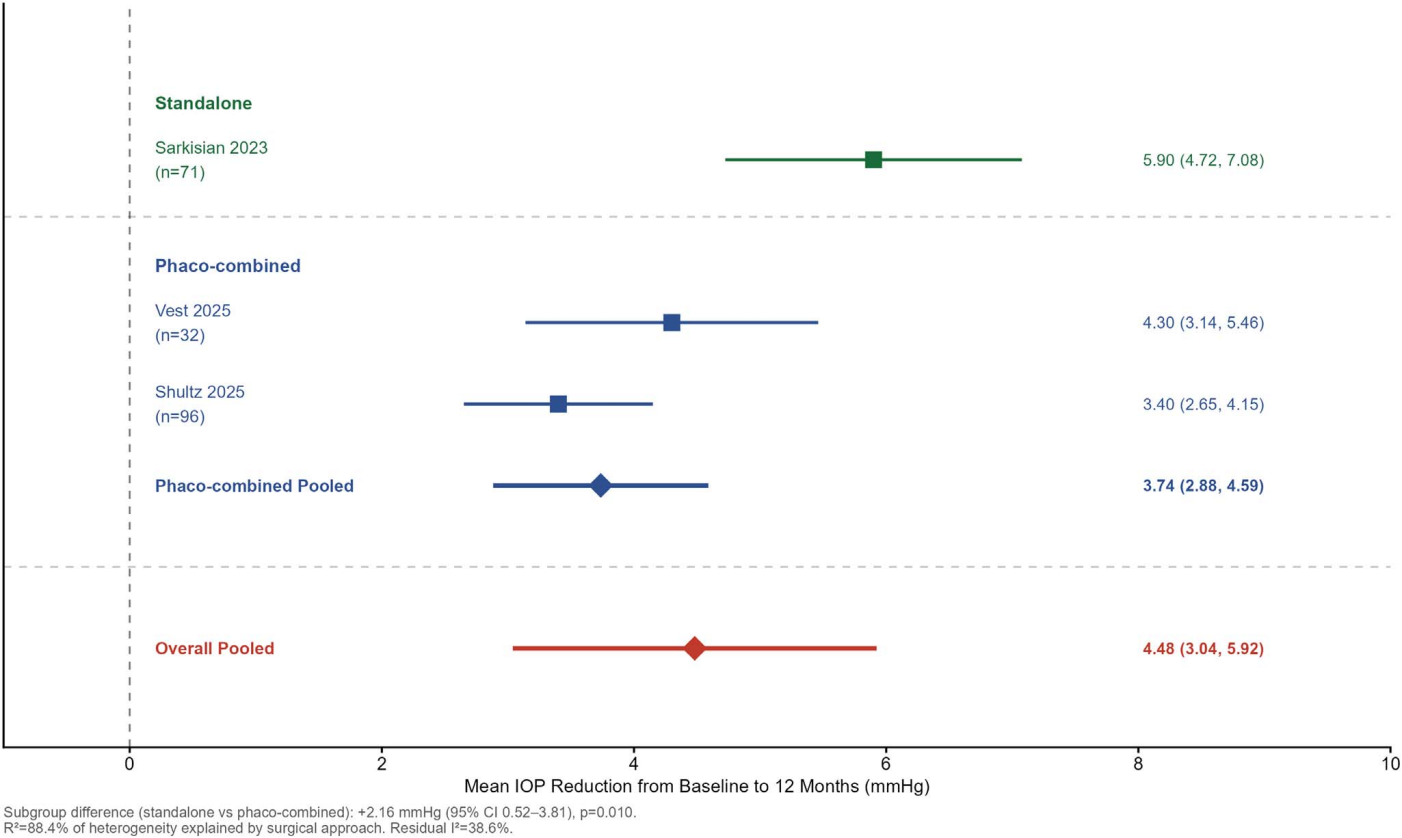
**Forest plot of mean intraocular pressure reduction (IOP) from baseline to 12 months following iStent infinite device implantation in 3 studies, stratified by surgical approach: stand-alone^25^ vs phaco-combined.^24,33^** Phaco-combined, iStent infinite device implantation combined with phacoemulsification.

A subgroup analysis comparing the stand-alone procedure vs phacoemulsification-combined procedure results showed a statistically significant difference in intraocular pressure reduction from baseline to 12 months (*P*=0.010) (Figure 4). The stand-alone approach (Sarkisian et al^25^) achieved a mean intraocular pressure reduction of 5.90 mm Hg, while the phacoemulsification-combined subgroup^24,33^ demonstrated a pooled mean estimate of 3.74 mm Hg intraocular pressure reduction. Meta-regression indicated that stand-alone implantation provided an additional 2.16 mm Hg reduction in intraocular pressure compared with the phacoemulsification-combined procedure (95% CI 0.52 to 3.81). Residual heterogeneity within the subgroup analysis was substantially reduced (I^2^=38.6%; QE=1.63; *P*=0.20), with QE representing the test statistic for residual heterogeneity in the mixed-effects meta-regression model and the nonsignificant *P* value indicating that any remaining unexplained heterogeneity after accounting for subgroup effects was not statistically significant.

### Primary Outcome: Medication Reduction

**Figure 5.**
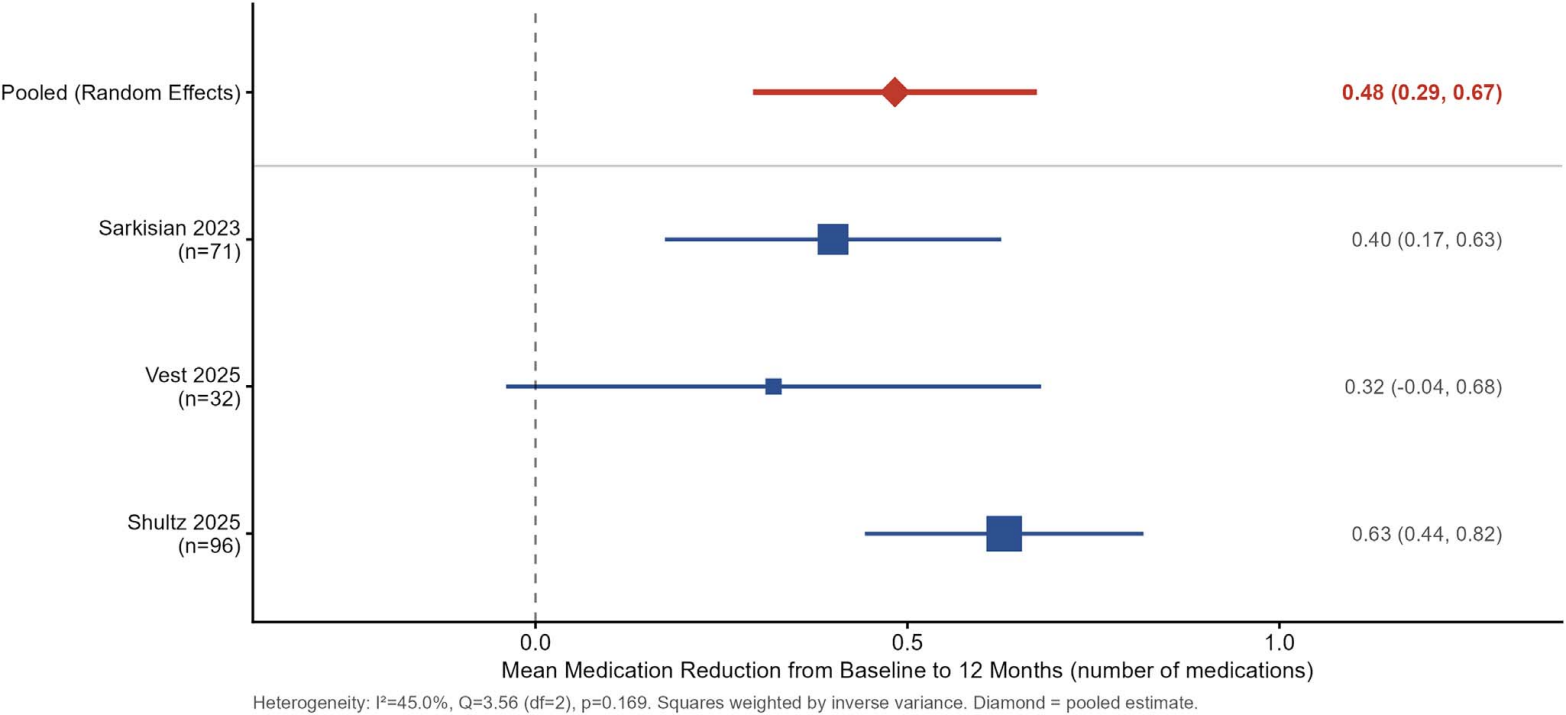
Forest plot of the mean reduction in number of medications from baseline to 12 months following iStent infinite device implantation in 3 studies.^24,25,33^

Pooled analysis demonstrated a mean reduction of 0.48 glaucoma medications (95% CI 0.29 to 0.67, *P*<0.0001) from baseline to 12 months (Figure 5). Individual study estimates ranged from a mean reduction of 0.32 medications (95% CI –0.04 to 0.68) in Vest et al^33^ to 0.63 medications (95% CI 0.44 to 0.82) in Shultz et al.^24^ Statistical heterogeneity was moderate (I^2^=45.0%; Q=3.56; *P*=0.169).

Subgroup comparison of medication reduction by surgical approach showed no statistically significant difference (*P*=0.65). The phacoemulsification-combined subgroup achieved a pooled medication reduction of 0.51 medications, while the stand-alone approach demonstrated a reduction of 0.40 medications. Meta-regression found no statistically significant subgroup effect (coefficient –0.11, 95% CI –0.60 to 0.38; *P*=0.65), and residual heterogeneity remained moderate (I^2^=55.5%; QE=2.25; *P*=0.13).

### Safety Outcomes

No cases of device removal or endophthalmitis were reported at 12 months. Secondary glaucoma interventions were required in 4.2% of eyes in Sarkisian et al,^25^ in 2.5% in Vest et al,^33^ and in 1.3% in Shultz et al.^24^ The most commonly reported adverse events in the pivotal trial (Sarkisian et al^25^) were intraocular pressure increase ≥10 mm Hg vs baseline (2.8%), loss of best-corrected visual acuity ≥2 lines (8.3%), ocular surface disease (9.7%), perioperative inflammation (6.9%), and visual field loss ≥2.5 decibels (6.9%). No loss of vision was considered device-related but was instead attributed to preexisting disease progression.^25^ Vest et al^33^ and Shultz et al^24^ reported no serious device-related adverse events in their retrospective cohorts.

## DISCUSSION

This systematic review with meta-analysis is the first quantitative synthesis of iStent infinite trabecular micro-bypass device outcomes at 12 months, encompassing 199 eyes from 3 studies. The pooled analysis shows clinically meaningful mean reductions in both intraocular pressure (4.48 mm Hg) and glaucoma medication burden (0.48 medications), with a favorable safety profile characterized by zero cases of device removal or endophthalmitis. Subgroup analysis shows stand-alone implantation achieved greater intraocular pressure reduction compared to phacoemulsification-combined procedures (an additional 2.16 mm Hg reduction; *P*=0.010), although medication reduction did not differ by surgical approach.

### Clinical Significance of Intraocular Pressure Reduction

The pooled intraocular pressure reduction of 4.48 mm Hg at 12 months represents a clinically meaningful treatment effect. Applying the framework from the Early Manifest Glaucoma Trial,^4^ the observed intraocular pressure reduction translates to an estimated 40% to 45% reduction in progression risk. This magnitude of effect compares favorably to traditional medical therapy, which can lower intraocular pressure but is limited by diminishing adherence and increasing treatment burden with polypharmacy.^8,9^

The differential efficacy between stand-alone (5.90 mm Hg) and phacoemulsification-combined (3.74 mm Hg) approaches merits careful interpretation. Cataract surgery alone can reduce intraocular pressure through mechanisms including improved aqueous outflow and changes in anterior chamber anatomy.^34^ In the phacoemulsification-combined group, the observed intraocular pressure reduction represents a combined effect of both the iStent infinite device and cataract surgery, so isolating the independent contribution of the device is difficult. The greater intraocular pressure reduction in the stand-alone group (5.90 mm Hg) likely reflects the device effect without confounding from phacoemulsification. Additionally, the stand-alone cohort enrolled patients with more refractory disease (baseline intraocular pressure of 23.4 mm Hg vs 17 to 18 mm Hg), suggesting greater potential for intraocular pressure reduction.

### Medication Reduction and Treatment Burden

A pooled medication analysis found a mean reduction of 0.48 glaucoma medications, which, while statistically significant, represents only approximately one-half medication per patient. This finding should be interpreted within the context of mean baseline medication burden that varied substantially across studies (range, 1.24 to 3.1 medications). In the stand-alone cohort with higher mean baseline medication use (3.1 medications), the reduction of 0.4 medications represents a 13% decrease from baseline. The phacoemulsification-combined studies that enrolled patients on fewer mean baseline medications (1.24 to 1.38 medications) demonstrated comparable absolute reductions in medication burden (range, 0.32 to 0.63 medications), although relative reductions were greater because of the baseline medication use.

From a clinical perspective, even modest medication reductions offer meaningful benefits. Adherence to topical glaucoma therapy declines precipitously with increasing medication burden, with studies demonstrating reduced adherence and increased barriers as regimen complexity increases.^11-13^ Moreover, chronic topical therapy carries cumulative risks of ocular surface disease and local adverse reactions, with potential for systemic absorption effects.^35^ The ability to reduce medication burden while maintaining or improving intraocular pressure control addresses an important challenge in glaucoma management.

### Safety Profile and Risk-Benefit Considerations

The data support a favorable short-term risk-benefit assessment of the iStent infinite device. The absence of serious complications (hypotony, device removal, endophthalmitis) and the low rates of secondary interventions (1.3% to 4.2%)^24,25,33^ contrast with traditional filtration surgery. Trabeculectomy and tube shunt procedures, while highly effective for intraocular pressure reduction, carry risks of hypotony, choroidal effusion, bleb-related infections, and vision-threatening complications.^15,16^ Adverse events were predominantly transient across all 3 studies.

Long-term data extending beyond 12 months will be essential to fully assess risks and benefits, as well as the durability of intraocular pressure control, late complications, and the need for subsequent interventions. The serious risk of bias identified in the 2 retrospective studies^24,33^ limits confidence in safety conclusions, as retrospective designs may underreport adverse events and lack systematic assessment protocols.

### Comparison to Other Micro-Invasive Glaucoma Surgery Devices

Outcomes from the iStent infinite device should be evaluated relative to other trabecular micro-invasive glaucoma surgery devices. The iStent infinite device was the first FDA-cleared trabecular micro-invasive glaucoma surgery device approved for stand-alone use, expanding treatment options for patients without concurrent cataract. The first-generation iStent and second-generation iStent inject, approved for use with concurrent phacoemulsification, demonstrated intraocular pressure reductions in pivotal clinical trials.^22,23^ However, direct comparison is limited by differences in study design, stand-alone status, baseline intraocular pressure, medication washout protocols, and the additive intraocular pressure-lowering effect of cataract surgery. The Hydrus Microstent (Alcon Inc), another trabecular device, demonstrated a mean reduction of 7.6 mm Hg in unmedicated intraocular pressure at 24 months when combined with cataract surgery in the HORIZON trial.^36^

### Heterogeneity and Study Limitations

The statistical heterogeneity for intraocular pressure reduction (I^2^=83.4%) was high; however, the subgroup analysis by surgical approach accounted for 88.4% of this heterogeneity, with residual heterogeneity substantially reduced (I^2^=38.6%) after accounting for stand-alone vs phacoemulsification-combined procedures. This finding suggests that the surgical approach is the primary source of variability in treatment effects. However, other unmeasured sources of heterogeneity include differences in glaucoma severity, surgical history, baseline medication burden, and surgeon experience.

Several limitations constrain the interpretation of these findings. First, the small number of studies limited our ability to assess publication bias and conduct comprehensive subgroup analyses. Second, the serious risk of bias in 2 of the 3 studies, attributable to the retrospective design and potential confounding, reduces confidence in effect estimates. Third, all studies were industry supported or conducted at centers with device expertise, potentially overestimating real-world effectiveness. Finally, lack of comparator groups in all 3 studies precludes assessment of the comparative effectiveness of the iStent infinite device vs medical therapy or other surgical interventions.

### Clinical Implications

For patients with glaucoma uncontrolled by medical therapy and without cataract, stand-alone iStent infinite device implantation is a low-risk alternative to trabeculectomy or tube shunt surgery, particularly for patients seeking to avoid the risks and postoperative burden of filtration surgery. However, because of the lack of randomized trial data, direct comparisons with less-invasive alternatives such as selective laser trabeculoplasty or simplified medical regimens are limited. Further study is needed to define the optimal place of iStent infinite device implantation in the glaucoma treatment algorithm.

### Future Research

Randomized controlled trials comparing the iStent infinite device to medical therapy or other micro-invasive glaucoma surgery devices would provide evidence for comparative effectiveness. Follow-up extending 3 to 5 years is essential to assess the durability of intraocular pressure control and determine if effect magnitude is maintained. Studies in diverse populations with varying glaucoma severities, subtypes beyond primary open-angle glaucoma, and different demographic groups would clarify the breadth of appropriate clinical applications.

## CONCLUSION

Implantation of the iStent infinite trabecular micro-bypass device resulted in clinically meaningful intraocular pressure and glaucoma medication reductions at 12 months with a favorable safety profile. Stand-alone implantation provided greater intraocular pressure reduction than phacoemulsification-combined procedures, consistent with higher baseline intraocular pressure in the stand-alone cohort and the intraocular pressure-lowering effect of cataract surgery. The evidence supports the iStent infinite device as an effective treatment option for open-angle glaucoma. However, the limited number of studies, risk of bias in retrospective cohorts, and short follow-up duration constrain definitive conclusions. Long-term randomized controlled trials are needed to establish comparative effectiveness and durability of treatment effects.

## Notes

*This article meets the Accreditation Council for Graduate Medical Education and the American Board of Medical Specialties Maintenance of Certification competencies for Patient Care, Medical Knowledge, and Practice-Based Learning and Improvement.*
